# Chemical Artificial Internalizing Receptors for Primary T Cells

**DOI:** 10.1002/advs.202001395

**Published:** 2020-07-26

**Authors:** Pere Monge, Anne Tvilum, Ane Bretschneider Søgaard, Kaja Borup Løvschall, Morten T. Jarlstad Olesen, Alexander N. Zelikin

**Affiliations:** ^1^ Department of Chemistry Aarhus University Langelandsgade 140 Aarhus C 8000 Denmark; ^2^ iNano Interdisciplinary Nanoscience Centre Aarhus University Langelandsgade 140 Aarhus C 8000 Denmark

**Keywords:** antibody–drug conjugates, artificial receptors, cell engineering, endocytosis

## Abstract

The newest generation of cell‐based technologies relies heavily on methods to communicate to the engineered cells using artificial receptors, specifically to deactivate the cells administered to a patient in the event of adverse effects. Herein, artificial synthetic internalizing receptors are engineered that function in mammalian cells in 2D and in 3D and afford targeted, specific intracellular drug delivery with nanomolar potency in the most challenging cell type, namely primary, donor‐derived T cells. Receptor design comprises a lipid bilayer anchor for receptor integration into cell membrane and a small xenobiotic molecule as a recognition ligand. Artificial receptors are successfully targeted by the corresponding antibody–drug conjugate (ADC) and exhibit efficient cargo cell entry with ensuing intracellular effects. Receptor integration into cells is fast and robust and affords targeted cell entry in under 2 h. Through a combination of the receptor design and the use of ADC, combined benefits previously made available by chimeric artificial receptors (performance in T cells) and the chemical counterpart (robustness and simplicity) in a single functional platform is achieved. Artificial synthetic receptors are poised to facilitate the maturation of engineered cells as tools of biotechnology and biomedicine.

## Graphical abstract

Artificial receptors are robust and simple (as small as 1 kDa), are installed into cells in under 2 h with minimal cell handling, and afford nanomolar potency of targeted drug delivery in primary T cells.

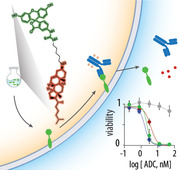

## 1 Introduction

Artificial internalizing and signaling receptors have become a highly warranted tool for biomedical sciences and specifically, for cell engineering. Cell‐based therapies are fast changing the landscape of biomedicine.^[^ ^1^, ^2^, ^3^, ^4^ ^]^ Indeed, cells can reconstruct tissues,^[^ ^5^ ^]^ deliver drugs across the blood‐brain barrier,^[^ ^4^, ^6^ ^]^ find and kill cancerous cells.^[^ ^7^, ^8^ ^]^ Nevertheless, these therapies are associated with significant risks of immunological and off‐target side effects. For safety, newest generations of engineered cells contain artificial “suicide switches,” such that administered cells can be deactivated in an occurrence of adverse events.^[^ ^8^, ^9^ ^]^ Highest success is envisioned when the switch is bio‐orthogonal, so as to provide cell‐specific effects in a complex multicellular environment,^[^ ^7^, ^10^, ^11^, ^12^ ^]^ and this is most successfully achieved using artificial receptors. Of these, chimeric artificial receptors^[^ ^10^, ^11^, ^12^ ^]^ are most successful to date but are highly complex by design and are highly labor intense to produce, and for engineering of donor‐derived cells, this process is highly expensive and lengthy in terms of time. In turn, artificial chemical receptors (that is, cell‐membrane anchored chemical tools acting as functional mimics of natural receptors)^[^ ^13^, ^14^, ^15^, ^16^, ^17^, ^18^, ^19^, ^20^ ^]^ are significantly simpler by structure, are robust, and for installation do not require extensive cell handling; but their performance is currently limited to the immortalized or cancerous cells and tissues, with fast metabolism and over‐active endocytosis.^[^ ^16^ ^]^ There is a high challenge for artificial endocytic receptors to achieve a combination of simplicity offered by chemical receptors with the broad scope and utility for chimeric counterparts, including receptor performance in the most challenging cell type, namely primary, donor‐derived T cells (as required for engineering of chimeric antigen receptor (CAR) T cells).

Herein we specifically address this challenge and describe chemical internalizing receptors that act as a “suicide switch” and afford nanomolar potency of receptor‐mediated drug delivery in primary, donor‐derived T cells. Toward the overall success, we engineer four artificial receptors to act as a pull‐in mechanism, and perform a structure‐activity screen in terms of receptor membrane localization and efficiency of cargo internalization. We then engineer corresponding antibody–drug conjugates (ADCs) and illustrate receptor‐specific cell killing in the nanomolar range of ADC, at concentrations that are safe to the receptor‐free cells. Most importantly, we demonstrate nanomolar potency in drug delivery to engineered primary T cells. The protocol for receptor installation into cells is fast (≈1 h), with minimal cell handling. Artificial chemical receptors presented in this work are simple by structure (as low as 1000 Da in molar mass) and by the protocol of cell engineering, and at the same time are highly powerful in performance. We believe that presented technology will prove to be significantly enabling and useful for cell engineering.

## 2 Results and Discussion

**Figure 1:**
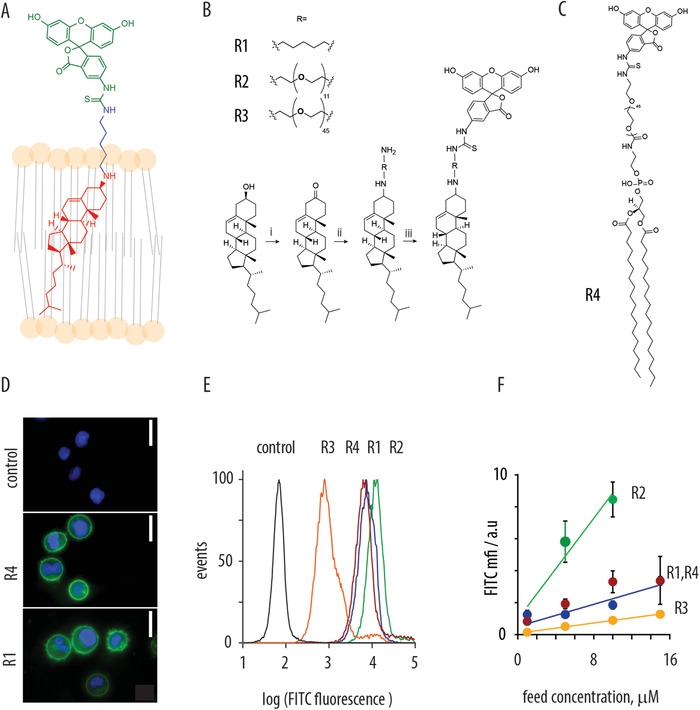
A) Schematic illustration of cholesterolamine–fluorescein conjugates acting as lipid‐bilayer‐anchored artificial recognition ligands; B) chemical synthesis of the cholesterolamine–fluorescein artificial receptors containing cadaverin or poly(ethyleneglycol diamine) spacer between the membrane anchor and the recognition ligand; reaction conditions: i) Dess–Martin Periodane (1.4 equiv.), H_2_O (1.1 equiv.) CH_2_Cl_2_, r.t., 60%; ii) Cadaverine HCl (4 equiv) or Boc‐PEG(*n* = 11 or 45), TEA (2 equiv.), Ti(OiPr)_4_ (1.32 equiv.), CH_2_Cl_2_:MeOH (1:1), r.t., then NaBH_4_ (1.2 equiv.), −78 °C; Boc‐deprotection for R2 and R3: TFA (200 equiv.), MeOH, 0 °C to r.t.; iii) FITC (1.2 equiv.), TEA (2.5 equiv.), C) artificial receptor based on a 1,2‐distearoyl‐sn‐glycero‐3‐phosphorylethanolamine (DSPE) lipid; D) fluorescence microscopy images of MOLT cells containing artificial receptor R1 and R4, scale bars: 10 µm; E) flow‐cytometry‐based quantification of cells fluorescence upon integration of artificial receptors R1–4; receptor feed concentration 4 × 10^−6^ m; F) quantified cells fluorescence upon integration of receptors R1–4 from solutions with varied receptor feed concentration.

For receptor design, we draw inspiration from the previous studies on cell surface engineering, specifically via membrane anchoring of the exofacial ligands.^[^ ^18^ ^]^ We chose this platform over metabolic labeling^[^ ^16^ ^]^ because the latter relies on a highly variable parameter, namely cell metabolism, and therefore can change dramatically from cell type to cell type (from model cells to primary cells, and from the fast‐proliferating cancer cells to the healthy counterpart). In contrast, membrane insertion is a fast, non‐specific mechanism, and is poised to be largely independent on the cell type. Learning from prior studies,^[^ ^18^, ^21^ ^]^ as a pull in mechanism for the artificial receptor, we chose to use cholesterolamine (**Figure** **1**A). This molecule proved to be highly efficient in membrane anchoring, advantageous in terms of persistence in the cell membrane over days of cell culture and over rounds of cell division and in recycling into the cell interior and back to the cell surface.^[^ ^18^, ^21^ ^]^ As a recognition ligand, we focused on a simple organic xenobiotic molecule, fluorescein. It has previously been used in a plethora of in vivo studies and to our knowledge documented no evidence of adverse effects (immune reaction etc).^[^ ^22^, ^23^ ^]^ This molecule is fluorescent, which greatly facilitates its visualization and quantification, and has a minimal permeability through the lipid bilayer of mammalian cells. The latter aspect is pivotal for the design of artificial receptors such that the recognition ligand remains exofacial and is accessible to the recognition events for drug targeting. Synthetic pathway to the smallest of artificial receptors was developed starting with oxidation of cholesterol into the corresponding ketone, followed by reductive amination using a diamine compound, cadaverine, and finally conjugation with fluorescein isothiocyanate, FITC (Figure 1B).We also synthesized artificial receptors using diamine derivatives of poly(ethylene glycol)s with molar mass of 500 and 2000 Da, so as to systematically vary the tether length between the recognition ligand and the membrane anchor. Finally, we also investigated fluorescein membrane anchoring using a two‐tailed lipid molecule (1,2‐distearoyl‐sn‐glycero‐3‐phosphorylethanolamine, DSPE, Figure 1C). For the latter, we used a commercially available DSPE‐PEG‐amine and directly conjugated it with FITC.

For installation into cells (MOLT4 cells, monocytic T cell derived cell line), receptors were administered as dimethylsulfoxide solutions onto cells with gentle vortexing. For each receptor molecule, this simple procedure resulted in efficient anchoring of amphiphilic molecules into the lipid bilayer of the cell, as visualized by fluorescence microscopy (Figure 1D). Cells fluorescence was quantified via flow cytometry, which indicated a narrow distribution of cells by the receptor content (Figure 1E). Fluorescence intensity expectedly increased with increased concentration of receptor molecules in the feed solution (Figure 1F). Interestingly, at matched concentrations, the four fluorescein‐containing molecules afforded different absolute levels of cell fluorescence, with receptor R2 being most fluorescent. This may indicate that the four molecules differ in their propensity to anchor into mammalian cells and/or result from a change in the fluorophore quantum yield of fluorescence due to microenvironment.

**Figure 2:**
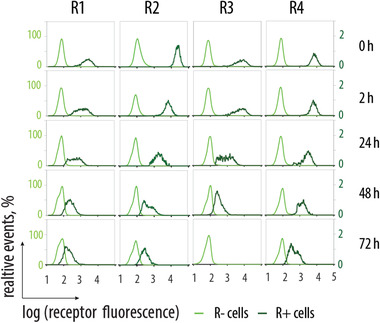
Flow‐cytometry‐based analysis of MOLT4 cells cultured as a 1:99 mixture of engineered (receptor‐equipped) to naïve (receptor‐negative) cells, over 72 h.

Association of cholesterol and DSPE with cell membranes is non‐specific and non‐covalent and in a mixed population of vesicles, cholesterol readily partitions between donor and acceptor lipid bilayers.^[^ ^24^ ^]^ Receptor “sharing” can decrease the ability to target engineered cells in circulation and therefore be unwanted, or have a beneficial effect and through receptor sharing, distribute artificial receptor molecules to the surrounding tissue. We quantified this phenomenon in cell culture using mixed populations of engineered (R+) and receptor‐naïve (R−) cells, at a 1% content of R+ cells. Over time in cell culture, the two cell populations (originally R+ and R−) converged by mean cell fluorescence value, a result which illustrates the expected receptor “sharing” (**Figure** **2**). Nevertheless, two cell populations could be reliably distinguished by intensity of fluorescence for at least 48 h in cell co‐culture, and for receptor R4 for at least 72 h.

**Figure 3:**
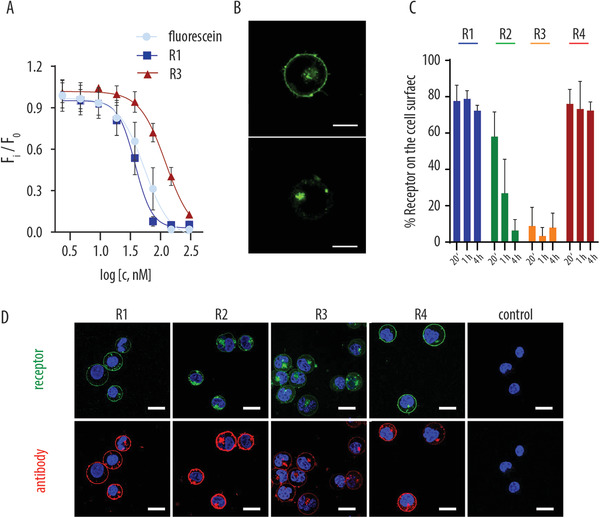
A) Quantitative data on fluorescence quenching in solutions of fluorescein or artificial receptors R1 and R3 in the presence of varied concentration of anti‐fluorescein antibody. Concentration of fluorescein, R1 and R3 was 0.1 × 10^−6^ m; phosphate buffered saline pH 7.4. B) Microscopy image showing fluorescence quenching of the artificial receptor integrated into the cell membrane lipid bilayer upon binding with an anti‐fluorescein antibody; scale bars: 5 µm; C) quantification of the fraction of receptor remaining on the cell surface over time; D) confocal laser scanning microscopy images of MOLT4 cells with integrated artificial receptors R1–4 upon targeting with rhodamine‐labeled anti‐fluorescein antibody, scale bars: 10 µm; control: no receptor cells + rhodamine‐labeled anti‐fluorescein antibody.

For targeting artificial receptors, we rely on anti‐fluorescein antibodies. Antibody binding to fluorescein is accompanied by a decrease of fluorescein fluorescence, and this phenomenon lends itself to quantify binding between the antibody and the antigen via facile fluorescence readout (**Figure** **3**A). In solution, dose dependent fluorescence quenching curves were rather similar for fluorescein and the receptor molecules derived thereof and reveal high affinity that characterizes the artificial antibody‐ligand pair used in this work. Fluorescence quenching was also observed upon antibody binding to the receptors integrated into lipid bilayers (Figure 3B), which can be used to quantify relative amounts of receptor molecules at cell surface (amenable to quenching by antibody) and within the cell interior (not subject to quenching by added antibody). Flow cytometry revealed that the four receptor molecules differed significantly in this readout. R3 molecules were largely confined to the cell interior, which was observed already at the earliest quantification time‐point (20 min). In turn, R2 molecules were gradually re‐distributed over time toward predominant intracellular localization (from 20 min to 4 h). Finally, R1 and R4 receptors were consistently found at cell surface in ≈75% quantity, and were amenable to antibody targeting. These data illustrate that a small variation in receptor design can lead to a pronounced change in the receptor properties, specifically in terms of cell membrane‐to‐interior distribution. This conclusion qualitatively agrees with prior publications on the subject^[^ ^25^ ^]^ and can be seen in both, variation of the exofacial hydrophilic tether (from R1 to R2 to R3) and variation of the membrane anchor (cholesterolamine versus DSPE, R3 and R4, respectively). Nevertheless, despite pronounced difference in the cell surface localization, each of the four receptors successfully mediated artificial endocytosis. Indeed, confocal laser scanning microscopy (CLSM) images illustrate that upon incubation of the rhodamine‐labeled anti‐fluorescein antibody with the MOLT4 cells containing artificial receptors, cells exhibit pronounced co‐localization of emission signals corresponding to fluorescein (receptor) and rhodamine (antibody) at the cell surface, Figure 3D. This phenomenon is not observed in control samples in which MOLT4 cells do not contain fluorescein and are incubated with the rhodamine‐labeled antibody, thus indicating successful, specific association of the antibody with the engineered cell surface. For each receptor, engineered cells also exhibit pronounced intracellular fluorescence corresponding to the internalized antibody. This observation is highly desired for successful drug targeting using artificial synthetic receptors developed herein.

**Figure 4:**
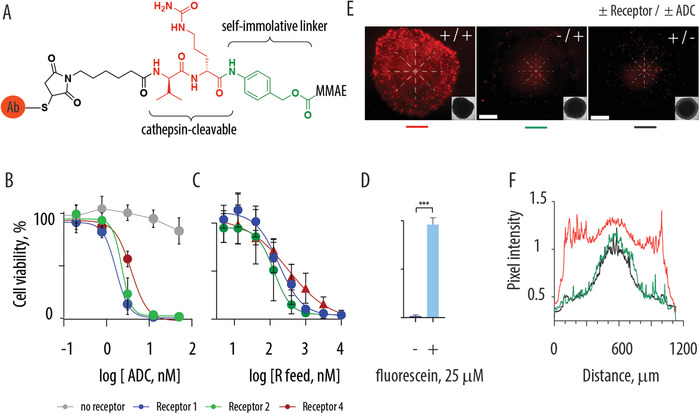
A) Schematic illustration of the antibody‐drug conjugate that contains a cathepsin‐sensitive scissile bond (valine‐citruline), a self‐immolative linker, and a conjugated drug, monomethyl auristatin E (MMAE); B,C) dose dependent toxicity of ADC in MOLT4 cells equipped with artificial synthetic receptors (R1,2,4) as a function of ADC concentrations ((B): receptors feed concentration 10 × 10^−6^ m) or as a function of receptor feed concentration ((C): ADC concentration 100 × 10^−9^ m) D) toxicity of fluorescein‐targeted ADC negated by an addition of soluble fluorescein; E) fluorescence microscopy images illustrating toxicity in HAP1 cell spheroids mediated by artificial synthetic receptors and the corresponding ADC. Images were obtained using propidium iodide live/dead staining; inserts show bright‐field microscopy images of the spheroids; scale bars: 200 µm. F) fluorescence profiles for spheroid specimen shown in (D). (B) and (C): results are based on at least three independent experiments and presented as mean ± S.D., statistical significance was evaluated using an un‐paired two‐tailed *t*‐test, *p* ≤ 0.001 (***).

For drug targeting, we synthesized antibody‐drug conjugates (ADC) on the template of the marketed ADC.^[^ ^26^ ^]^ Specifically, valine‐citrulline was used a cleavable linker, and monomethyl auristatin E (MMAE) was chosen as an ultrapotent conjugated toxin (**Figure** **4**A). Fluorescein‐targeted ADC were administered onto the engineered MOLT4 cells, and cell viability was quantified 72 h later. Dose response curves demonstrated that ADC were highly potent and elicited efficient cell killing at concentrations that are safe to cells without the engineered receptor (Figure 4B). The structure–activity relationship reveals that three out of four receptors were rather similar in their ability to facilitate receptor mediated cell entry for the ADC, whereas the cholesterolamine‐PEG_2000_ receptor (R3) was not effective. The most important observation from this data is that the synthetic artificial receptors in MOLT4 cells afforded nanomolar potency of cell killing (1.6 ± 0.2, 2.2 ± 0.7, and 3.2 ± 0.8 × 10^−9^ m for R1, R2 and R4, respectively). This potency exceeds by several orders of magnitude the levels reported previously for other types of synthetic receptors (typically characterized with micromolar potency^[^ ^17^, ^19^ ^]^) and, most importantly, is on par with drug delivery targeted to natural receptors. We also quantified cell killing in a dose dependent manner with regards to the feed concentration of receptor molecules administered onto cultured cells (Figure 4D). To this end, MOLT4 cells were incubated with increasing concentrations of receptor molecules R1, R2 or R4, and thereafter with the ADC taken at 100 × 10^−9^ m. These experiments reveal that receptor molecules performed rather similarly in this readout, with only minor difference in the apparent EC50 values (< twofold variation). In each case, successful cell engineering toward artificial endocytosis was achieved at sub‐micromolar concentrations of receptor molecules. As the last element of proof for receptor mediated activity of ADC, we used soluble fluorescein and observed that this measure precludes activity of ADC, as would be expected for a competitive saturation of the antibody by the soluble ligand (Figure 4D).

Performance of artificial receptors was further illustrated in cancer cell spheroids, that is, at conditions employed to better reflect the 3D tissue organization.^[^ ^27^ ^]^ Specifically, spheroids were grown using HAP1 liver cancer cells. Receptor R1 alone or ADC administered onto spheroids had negligible change in the spheroid cell viability. In both cases, spheroids exhibited a characteristic necrotic core and live cells comprising the spheroid periphery (Figure 4E). In contrast, spheroid that was exposed to a solution of artificial receptor R1 and subsequently to the ADC exhibited substantial cell death, observed throughout the spheroid volume. Visual observations were validated by quantitative image analysis (Figure 4F). Taken together, results in Figure 4 illustrate that artificial receptors based on cholesterolamine or DSPE as a pull‐in mechanism and fluorescein as the recognition ligand potently mediate cell entry for targeted ADC with ensuing intracellular effects, and this is observed in 2D and in 3D cell culture.

**Figure 5:**
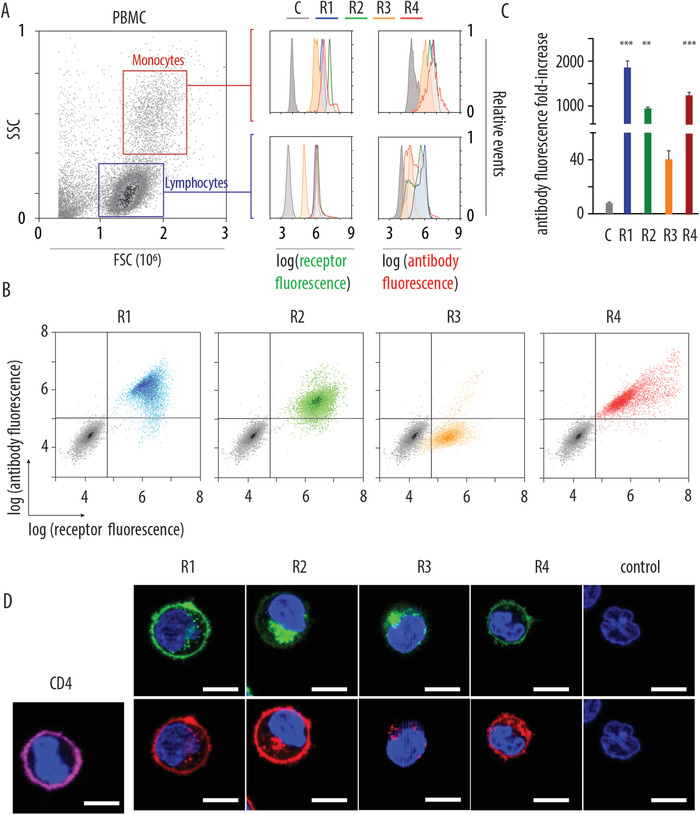
A,B) Flow cytometry dot‐plot analysis of fluorescence for peripheral blood mononuclear cells (A) or purified CD4+ T cells (B) incubated with solutions of artificial receptors R1–4 and subsequently with rhodamine‐labeled anti‐fluorescein antibody; in (B), control cells not exposed to receptor solutions are presented in grey, receptor‐positive cells are color coded in blue, green, orange, and red for R1–4, respectively; C) fold‐increase in fluorescence corresponding to the antibody targeted to the artificial synthetic receptors R1–4 integrated into the CD4+ T cells; results are based on three independent experiments and presented as mean ± S.D., statistical significance was evaluated using a two‐way ANOVA with Sidak's multiple comparisons test, *p* ≤ 0.01 (**), *p* ≤ 0.001 (***). D) Confocal laser scanning microscopy images demonstrating internalization of the rhodamine‐labeled antibody targeted to the artificial synthetic receptors R1–4 integrated into the CD4+ T cells, as well as CLSM image illustrating predominantly surface‐bound fluorescent antibody targeted to the natural CD4 receptor on the T cells, scale bars 10  µm.

Encouraged by the above data, we next aimed to investigate the utility of synthetic artificial internalizing receptors in primary, donor derived white blood cells. Monocytes and lymphocytes are among the most warranted cells in the context of cell engineering and cell‐based therapies, and dedicated communication route to these cells would facilitate their biomedical use.^[^ ^28^ ^]^ Pooled peripheral blood mononuclear cells from human donors were mixed with solutions of artificial receptors R1–4, and analyzed on flow cytometry. Primary gating was conducted based on light scattering parameters (forward and side scattering) to identify monocytes and lymphocytes, for independent quantification of fluorescence within these cells sub‐types. For each receptor and for each cell population, we observed full population shift and narrow distribution of cells by fluorescence signal, **Figure** **5**A. Quantitatively, much similarly to the data from MOLT4 cells, we observed that receptor R3 afforded lowest cell fluorescence, which was true for both, monocyte and lymphocyte cell populations. Most importantly, in each case, monocytes and lymphocytes equipped with artificial receptor molecules were successfully targeted by rhodamine‐labeled anti‐fluorescein antibody, as evidenced by flow cytometry analysis for the cell‐associated rhodamine fluorescence.

Next, we isolated the CD4+ subset population of T cells, which is notoriously resilient to the intracellular drug delivery endeavours, in large part due to the non‐internalizing character of the CD4 receptor.^[^ ^29^ ^]^ CD4+ T cells from donor derived blood were exposed to solutions of artificial receptors R1–4, and subsequently to the rhodamine‐labeled anti‐fluorescein antibody. Fluorescence of cells was analyzed via flow cytometry, Figure 5B. For each artificial receptor type, we observed a significant increase in fluorescence corresponding to receptor molecules (control cells not exposed to receptor solutions are presented in grey, receptor‐positive cells are color coded in blue, green, orange, and red for R1–4, respectively). Fluorescent signal of the targeted antibody showed corresponding increase for R1, R2, and R4 (but not for R3 receptor). Quantitatively, we observed at least a 1000‐fold increase in fluorescence upon targeting artificial synthetic receptors with the corresponding antibody (except for the R3 receptor in which case fold‐difference in fluorescence was markedly lower, ≈40‐fold), Figure 5C. CLSM was employed to illustrate that artificial receptor targeting in CD4+ T cells leads to internalization of the targeted antibody, Figure 5D. Indeed, representative images illustrate that a significant proportion of the fluorescent signal corresponding to the targeted antibody is observed within the cells, indicating cell entry for the antibody. This comes in contrast with the fluorescent signal for the antibody targeted to the natural CD4 receptor, in which case we observe predominantly surface‐bound antibody with minimal if any fluorescent signal within cells. Minimal internalization of the CD4‐targeted antibody agrees well with prior reports on the subject^[^ ^29^ ^]^ and further highlights the appeal of synthetic artificial receptors developed in this work. Artificial receptors afford efficient targeting of the engineered T cells as well as cell entry for the targeted cargo, as is required for intracellular drug delivery. Highly important is that T cell engineering as performed herein is very fast and the overall procedure takes under 2 h for the entire process, from receptor installation to the registered antibody engagement with the engineered synthetic receptor.

**Figure 6:**
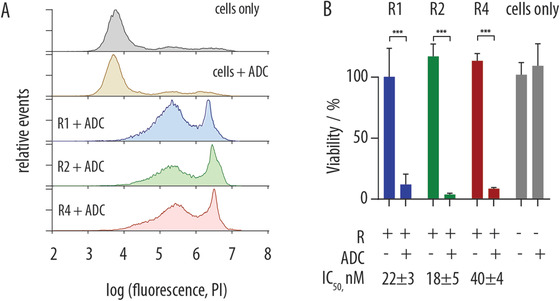
Flow‐cytometry‐based read out for cytotoxicity of ADC targeted to the artificial synthetic receptors R1–4 integrated into the donor derived primary CD4+ T cells: A) histogram view for the propidium iodide live/dead staining for cells following incubation with ADC (150 × 10^−9^ m) and additional 48 h in cell culture; B) corresponding end‐point read‐out values for cell viability, as well as the IC_50_ values for ADC‐mediated toxicity in CD4+ T cells equipped with artificial synthetic receptors R1, R2, and R4. Results are based on three independent experiments and presented as mean ± S.D., statistical significance was evaluated using a two‐way ANOVA with Sidak's multiple comparisons test, *p* ≤ 0.001 (***).

Finally, to demonstrate specific drug targeting to the primary T cells, we used the MMAE‐containing ADC targeted to fluorescein. Upon incubation with the antibody (150 × 10^−9^ m expressed in equivalent MMAE content) and following additional 48 h in culture, engineered T cells were stained with propidium iodide as a live/dead stain. Flow cytometry observation demonstrates complete cell killing for the engineered receptor‐containing cells, whereas naïve cells exposed to this ADC concentration exhibit viability identical to the non‐treated control cells, **Figure** **6**A. Of highest importance, receptor incorporation into T cells, in absence of ADC, elicited no detrimental effect on the cell viability (Figure 6B). In contrast, ADC targeting produced strong cytotoxic effects mediated by artificial receptors, and dose response studies revealed that potency of ADC in primary T cells was in the nanomolar range.

Taken together, results of this study demonstrate that synthetic artificial internalizing receptors can be engineered as a powerful tool for biotechnology and biomedicine, and specifically for intracellular drug delivery into some of the most challenging cell types such as primary T cells. The developed methodology relies on antibody targeting, aimed at a synthetic cell surface marker, whereby the recognition ligand is a xenobiotic, orthogonal to all the natural receptors in the body. ADC targeted to the artificial receptor revealed therapeutic responses in 2D and in 3D cell culture and in primary, donor derived T cells, with nanomolar potency. The protocol for cell surface engineering is robust and fast, and within 2 h it is possible to achieve intracellular drug delivery mediated by synthetic receptors. Current work is underway to design optimized artificial receptors with enhanced persistence in the cell membrane and decreased “sharing” of receptor molecules between cells, so as to engineer cells that can be targeted over extended times in circulation. We strongly believe that artificial synthetic receptor methodology will prove highly useful in the era of cell engineering and cell‐based therapies.

## Conflict of Interest

The authors declare no conflict of interest.

## Supporting information

Supporting Information
