# Supplementary material for: Chemical Artificial Internalizing Receptors for Primary T Cells
Source: Adv Sci (Weinh). 2020 Jul 26;7(18):2001395. doi: 10.1002/advs.202001395 (PMC7509642; doi:10.1002/advs.202001395)

Copyright WILEY-VCH Verlag GmbH & Co. KGaA, 69469 Weinheim, Germany, 2020.

ADVANCED SCIENCE
Open Access

Supporting Information

for *Adv. Sci.*, DOI: 10.1002/advs.202001395

Chemical Artificial Internalizing Receptors for Primary T Cells

*Pere Monge, Anne Tvilum, Ane Bretschneider Søgaard, Kaja Borup Løvschall, Morten T. Jarlstad Olesen, and Alexander N. Zelikin**

Copyright WILEY-VCH Verlag GmbH & Co. KGaA, 69469 Weinheim, Germany, 2020.

Supporting Information

Chemical artificial internalizing receptors for primary T cells

*Pere Monge, Anne Tvilum, Ane Bretschneider Søgaard, Kaja Borup Løvschall, Morten T. Jarlstad Olesen, Alexander N. Zelikin**

I. Synthetic Methods: 2

II. Biological methods 14

III. ^{1}H and ^{13}C NMR spectra 23

I. Synthetic Methods:

General

All chemicals were purchased from Sigma-Aldrich and used without further purification. DSPE-PEG(2000) amines was purchased from Laysan Bio. Boc-NH-PEG(2000) amine was purchased from Biochempeg. MMAE was purchased from Apichem, antiFITC antibody was purchased from ThermoFischer. MC-Val-Cit-PABC-PNP was purchased from IRIS Biotech. Deuterated Solvents were purchased from Euriso-Top. All moisture and air sensitive reactions were performed in flame dried glassware under a positive pressure of argon of N_2. Dichloromethane (CH_2Cl_2) was dried over aluminum oxide via an MBraun SPS-800 solvent purification system. Dimethylformamide (DMF) and trimethylamine (TEA) was purchased in an anhydrous state from Sigma-Aldrich. Thin layer chromatography (TLC) analysis was carried out on silica coated aluminum foil plates (Merck Kieselgel 60 F254). The TLC plates were visualized by UV irradiation and/or by staining with $KMnO_4$ and ninhydrin stain. Flash column chromatography was carried out using silica gel – high purity grade (w/Ca, ~0.1%, 230-400 mesh particle size, 60Å pore size) as the stationary phase acquired by Sigma-Aldrich. Mass spectra (High Resolution Mass Spectrometry – HRMS) were recorded on a Micromass LC-TOF spectrometer with positive electrospray ionization.

MADLI-TOF-MS spectra of the antibody-drug conjugates (ADC) was carried out on a MALDI-TOF-MS spectrometer (Autoflex; Bruker Daltonics) with the FLEX control software. The matrix used for the ADC's was prepared in the following way: to a 1:1 mixture of MQ and MeCN 0.1 % TFA and 20 mg/ml sinapinic acid was added. The samples for analysis was prepared as follow; a mixture containing the ADC's (1 mg/ml) was mixed with the matrix in a 1:1 ratio on a MTP anchor chip (Bruker Daltonik, Bremen, Germany) and allowed to air dry for cocrystallization.

Nuclear magnetic resonance (NMR) spectra were recorded on a Bruker Ascend 400 spectrometer, running at 400 and 101 MHz for ^{1}H and ^{13}C, respectively. Chemical shift are reported in ppm relative to the residual solvent signals: chloroform 7.26 ppm and 77.16 ppm, methanol 3.31 ppm, DMSO 2.50 ppm. Multiplicities are indicated using the following abbreviations: s=singlet, d=doublet, t=triplet, q=quartet, m=multiplet. Preparative HPLC purifications were carried out on an Agilent 1260 Infinity II with a Luna C18 column with particle size at 5 μm, a length at 250 mm and an internal diameter of 10 mm from phenomenex. Mobile phases were ultrapure water (MQ) and acetonitrile supplemented with 0.1 % TFA (v/v). MQ was received from Mili Q direct 8 system (Milipore). Analytical HPLC analysis was carried out an Agilent 1260 Infinity II with an EC-C18 column with particle size 2.7 μm, a length at 100 mm and an internal diameter of 4.6 mm. The mobile phases were the same as described for prepHPLC purifications.

General Boc-deprotection. Boc-protected amine (0.05 mmol, 1 equiv.) was dissolved in MeOH (1 mL) and cooled to 0 ^{o}C. TFA (10 mmol, 200 equiv.) was added and the reaction was left at r.t. for 5 h, followed by concentration. The isolated material was used without further purification.

Synthesis of a

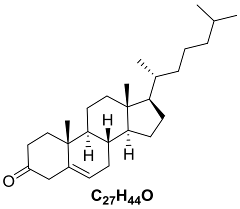

$C_{27}H_{44}O$
a

Cholesterol (1.063 g, 2.75 mmol, 1 equiv.) and DMP (1.5478 g, 3.65 mmol, 1.4 equiv.) were dissolved in CH_2Cl_2 (30 ml). H_2O (51.3 μL, 2.85 mmol, 1.1 equiv.) was mixed well with CH_2Cl_2 (51.3 mL) and added dropwise to the reaction mixture. After 25 min. the solution was washed twice with a 1:1 mixture containing a 10 % $Na_2S_2O_3$ aqueous solution and a saturated $NaHCO_3$ solution (50 mL), followed by washing with water (50 mL) and brine (50 mL). The aqueous phases were extracted with CH_2Cl_2, followed by washing of the organic phase with water and brine. The organic phases were combined, dried over $NaSO_4$ and concentrated *in vacou*.

The crude product was purified by flash column chromatography (Pentane to 2 % EtOAc in pentane), yielding the product as a white powder (0.565 g, 1.546 mmol, 60 %).

$\mathbf{R_f}$ (pentane: 2% EtOAc) = 0.263

^{1}H-NMR: (400 MHz, Chloroform-d) δ 5.34 (s, 1H), 3.28 (dd, J = 16.9, 3.0 Hz, 1H), 2.82 (dd, J = 16.6, 2.3 Hz, 1H), 2.48 (td, J = 14.6, 13.8, 5.8 Hz, 1H), 2.36 – 2.22 (m, 1H), 2.14 – 1.73 (m, 4H), 1.18 (s, 3H), 1.55-0.99 (m, 22H), 0.92 (d, J = 6.5 Hz, 3H), 0.86 (dd, J = 6.6, 1.8 Hz, 6H), 0.71 (s, 3H)

^{13}C NMR (101 MHz, Chloroform-*d*) δ 199.89, 171.95, 123.88, 56.21, 56.00, 53.93, 42.51, 39.75, 39.63, 38.74, 36.24, 35.89– 35.74 (m), 34.14, 33.10, 32.17, 28.32, 28.16, 24.32, 23.94, 22.97, 22.71, 21.15, 18.77, 17.52, 12.09

HRMS (ESI+) *m/z* calculated (calcd.) for $C_{27}H_{44}O + H^+$: 385.3466; found: 385.3456, calcd. for $C_{27}H_{44}O + Na^+$: 407.3285; found: 407.3259, calcd. for $2C_{27}H_{44}O + Na^+$: 791.6679; found: 791.6665

Synthesis of b.1

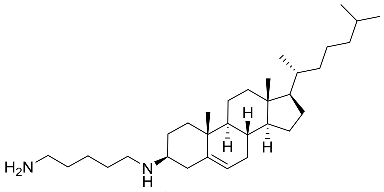

$C_{32}H_{58}N_2$
b.1

Cadaverine hydrochloride (110.9 mg, 1.09 mmol, 4 equiv.) was dissolved in dry MeOH (2 mL) followed by addition of TEA (76 μL, 0.568 mmol, 2 equiv.). 4-cholesten-3-one (a) (112.4 mg, 0.292 mmol, 1 equiv.) and Ti(i-OPr)$_4$ (102 μL, 0.343 mmol, 1.32 equiv.) was dissolved in a mixture of dry MeOH (1 mL) and CH_2Cl_2 (1 mL) and subsequently added dropwise to the mixture containing cadaverine hydrochloride. The reaction was stirred for 24 h. under N_2 followed by cooling to -78 ℃ and addition of $NaBH_4$ (12.6 mg, 0.333 mmol, 1.2 equiv.). After 2 h. the reaction was quenched with H_2O (2 mL) and stirred for another 20 min. followed by filtration over a celite path, washed with CH_2Cl_2 and MeOH, dried with Na_2SO_4 and concentrated.

The crude was purified with preparative HPLC (30 % MeCN to 100 % MeCN over 15 min., 100 % MeCN to 20 min, 30 % MeCN to 22 min), with a retention time of 11.5 min. yielding a white powder (44.2 mg, 0.26 mmol, 36 %).

^{1}H NMR (400 MHz, MeOD-*d*$_4$) δ 5.32 (s, 1H), 3.75-3.66 (m, 1H), 3.04 (d, *J* = 8.1 Hz, 2H), 2.94 (t, *J* = 7.7 Hz, 2H), 2.35-2.22 (m, 1H), 2.19-1.99 (m, 3H), 1.94 – 0.75 (m, 55H),

^{13}C NMR (101 MHz, MeOD) δ 154.29, 115.06, 57.56, 57.44, 56.31, 55.84, 45.18, 43.62, 41.12, 40.68, 40.31, 38.60, 37.33, 37.09, 36.29, 34.09, 33.48, 29.26, 29.15, 28.01, 27.00, 25.19, 24.93, 24.48, 23.51, 23.19, 22.95, 22.16, 19.18, 19.13, 12.41.

HRMS (ESI+) *m/z* calculated (calcd.) for $C_{32}H_{58}N_2 + H^+$: 471.4673; found: 471.4683, calcd. for $C_{32}H_{58}N_2$+H^++Na: 247.2282; found: 247.2412, calcd. for $C_{32}H_{58}N_2$+2Na: 258.2192, found: 258.2347

Synthesis of b.2

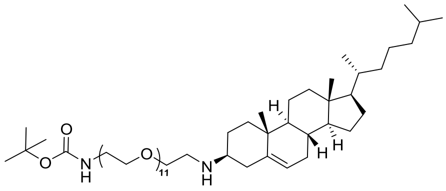

$C_{56}H_{104}N_2O_{13}$
b.2

Boc-Peg-NH_2 (211.4 mg, 0.328 mmol, 1.3 equiv.) was dissolved in dry MeOH (2 mL) followed by addition of TEA (68.9 μL, 0.496 mmol, 2 equiv.). 4-cholesten-3-one (**a)** (101.5 mg, 0.265 mmol, 1 equiv.) and Ti(i-OPr)$_4$ (103 μL, 0.348 mmol, 1.32 equiv.) was dissolved in a mixture of dry MeOH (1 mL) and CH_2Cl_2 (1 mL), and subsequently added dropwise to the mixture containing Boc-Peg-NH_2. The reaction was stirred for 24 h. under N_2 followed by cooling to -78 °C and addition of $NaBH_4$ (12.6 mg, 0.333 mmol, 1.2 equiv). After 2 h. the reaction was quenched with H_2O (2 mL) and stirred for another 20 min. followed by filtration of the crude over a celite path, washed with CH_2Cl_2 and MeOH, dried with Na_2SO_4 and concentrated.

The crude was concentrated and purified with flash column chromatography CH_2Cl_2 to 10 % MeOH in CH_2Cl_2, yielding the product as a yellow oil (77.6 mg, 0.248mmol, 31 %).

^{1}H NMR (400 MHz, Chloroform-*d*) δ 5.38 (s, 1H), 3.90 – 3.74 (m, 4H), 3.74 – 3.54 (m, 71H), 3.51 (t, *J* = 5.2 Hz, 4H), 3.32 – 3.24 (m, 3H), 3.07 (t, *J* = 5.3 Hz, 2H), 2.26 – 0.52 (m, 88H).

HRMS (ESI+) *m/z* calculated (calcd.) for $C_{56}H_{104}N_2O_{13}$ + H^+: 1013.7573; found: 1013.7607, clalcd. for $C_{56}H_{104}N_2O_{13}$+CAN+$2H^+$: 527.8956; found: 527.3656

Synthesis of b.3

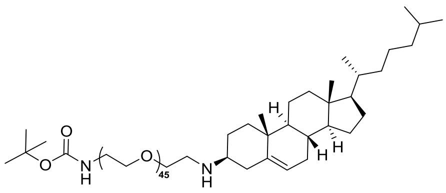

$C_{124}H_{240}N_2O_{47}$
b.3

Boc-NH-Peg(2kDa)-NH_2 (179.3 mg, 0.0836 mmol, 1.2 equiv.) was dissolved in dry MeOH (2 mL) followed by addition of TEA (18.1 μL, 0.13 mmol, 2 equiv.). 4-cholesten-3-one (26.4 mg, 0.0686 mmol, 1 equiv.) and Ti(O-iPr)$_4$ (25.4 μL, 0.0858 mmol, 1.32 equiv.) was dissolved in dry MeOH (1 mL) and dry CH_2Cl_2 (1.5 mL), followed by dropwise addition to the mixture containing Boc-NH-Peg(2kDa)-NH_2. After 15 h. the reaction was cooled to -78 ℃ followed by addition of $NaBH_4$ (3.2 mg, 0.046 mmol, 1.3 equiv.). After 2 h. the reaction was quenched with H_2O (3 mL) and stirred for another 20 min. The precipitate was removed with centrifugation and washed with EtOAc and MeOH twice. The organic phases were combined, dried with Na_2SO_4, filtered and concentrated.

The crude was purified with flash column chromatography (CH_2Cl_2 to 10 % MeOH in CH_2Cl_2), followed by precipitation in cold ether yielding the product as a yellow oil (94 mg, 0.037 mmol, 58 %).

^{1}H NMR (400 MHz, Chloroform-*d*) δ 5.43 (s, 0.4H), 4.38 – 3.01 (m, 254H), 2.33 – 0.67 (m, 47H).

Synthesis of R1

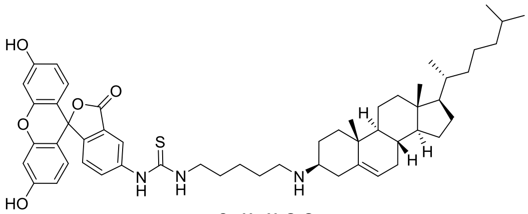

$C_{53}H_{69}N_3O_5S$
R1

b.1 (15.4 mg, 0.0327 mmol, 1 equiv.) was dissolved in MeOH (1 ml) followed by addition of TEA (8.26 μl, 0.0594 mmol, 2 equiv.). Flourescein isomer I (11.6 mg, 0.0297 mmol, 1.2 equiv.) was dissolved in 1:1 MeOH:DMF (1 ml) and added dropwise to the mixture containing b.1. The reaction was left stirring in the dark for 4 h., concentrated, re-dissolved in CH_2Cl_2 and washed with brine and water. The organic phase was dried with sodium sulfate and concentrated.

The crude was purified with preparative HPLC (10 % MeCN to 100 % MeCN from 0 to 15 min, 100 % MeCN from 15 min. to 19 min, 100 % MeCN to 10 % MeCN from 19 min to 20 min.) with a retention time of 15 min. yielding the product as a yellow powder (2.4 mg, 0,003 mmol, 9 %).

^{1}H NMR (400 MHz, MeOD-d_4) δ 8.15 (s, 1H), 7.76 (d, *J* = 7.6 Hz, 1H), 7.20 (d, *J* = 8.3 Hz, 1H), 6.91 – 6.50 (m, 6H), 5.32 (s, 1H), 3.71 (s, 3H), 3.17 – 3.03 (m, 2H), 2.30 (t, *J* = 13.7 Hz, 1H), 2.18 – 0.61 (m, 59H).

HRMS (ESI+) *m/z* calculated (calcd.) for $C_{53}H_{69}N_3O_5S + H^+$: 860.5030; found: 860.5044, calcd. for $C_{53}H_{69}N_3O_5S$ + 3ACN + $2H^+$: 492.2950; found: 492.1590, calcd. for $C_{53}H_{69}N_3O_5S + 2\ H^+$: 430.7552; found: 430.7547

Synthesis of R2

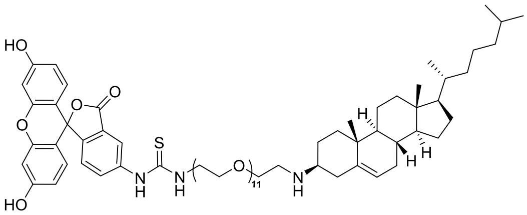

$C_{72}H_{107}N_3O_{16}S$
R2

b.2 was boc deprotected as described in *general method 1.*

Deprotected **b.2** (45.1 mg, 0.0494 mmol, 1 equiv.) was dissolved in dry DMF (2 mL) followed by addition of TEA (17.2 μL, 0.123 mmol, 2.5 equiv.) under N_2, followed by addition of Fluorescein isomer I (23.1 mg, 0.0593 mmol, 1.2 equiv.). After 8 hours the crude was concentrated and purified with preparative HPLC (10 MeCN to 100 % MeCN over 15 min., 100 % MeCN to 19 min., 10 % MeCN to 20 min and from 20-25 min.) with a retention time of 15 min. yielding the desired product as a yellow oil (3.4 mg, 0.003 mmol, 5 %)

^{1}H NMR (400 MHz, DMSO-d_6) δ 8.23 (s, 1H), 7.81 (d, *J* = 8.3 Hz, 1H), 7.17 (d, *J* = 8.1 Hz, 2H), 6.76 – 6.44 (m, 2H), 5.32 (s, 2H), 3.86 – 3.44 (m, 19H), 2.37 – 0.62 (m, 5H).

HRMS (ESI-) *m/z* calculated (calcd.) for $C_{72}H_{107}N_3O_{16}S$ - H^+: 1300.7299; found: 1300.6158, calcd. for $C_{72}H_{107}N_3O_{16}S$ - $2H^+$: 649.8613; found: 649.8722, calcd. for $C_{53}H_{69}N_3O_5S$ + 2 H^+ : 430.7552; found: 430.7547

Synthesis of R3

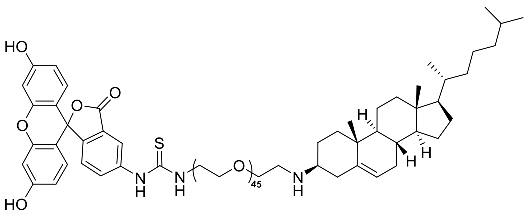

$C_{140}H_{243}N_3O_{50}S$
R3

b.3 was boc-deprotected as described in *general method 1.*

Deprotected **b.3** (28.9 mg, 0.012 mmol, 1 equiv.) was dissolved in DMF (2 mL) and TEA (2 uL) was added. FITC (5.4 mg, 0.0139 mmol, 1.1 equiv.) was dissolved in DMF (1 mL) followed by addition to the reaction mixture. The reaction mixture was stirred for 3.5 hours in dark, followed by concentration *in situ*.

The crude product was dissolved in 30 % MeOH in H_2O and added to a dialysis cassette (MWCO: 2 kDa, *slide-A-Lyzer membrane*, 0.5-3 mL, regenerated cellulose membrane) and submerged to a dialysis solution containing 10 % MeOH in H_2O for 20 hours. Afterwards the solution was freeze dried yielding a yellow powder (25 mg, 0.009 mmol, 71 %).

^{1}H NMR (400 MHz, Chloroform-*d*) δ 9.58 (s, 1H), 8.11 (s, 1H), 7.07 (d, *J* = 8.3 Hz, 1H), 6.86 – 6.48 (m, 5H), 5.34 (s, 1H), 3.97 – 3.35 (m, 256H), 2.36 – 0.53 (m, 37H).

MALDI-TOF MS (m/z) calculated for $C_{140}H_{245}N_3O_{50}S$: 2801.65; found: 2801.10

Synthesis of R4

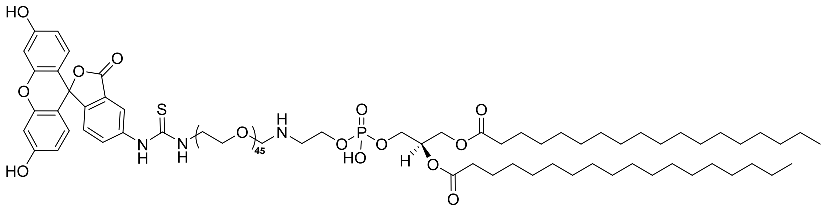

$C_{150}H_{270}N_3O_{58}PS$
R4

DSPE-Peg-NH_2 (52.9 mg, 0.019 mmol, 1 equiv.) was dissolved in dry CH_2Cl_2 (1 ml) followed by addition of TEA (3.74 μL, 0.0268 mmol, 1.5 equiv.). FITC (9.0 mg, 0.0231 mmol, 1.2 equiv.) was dissolved in dry DMF (0.2 ml) and added to the reaction mixture containing DSPE-Peg-amine. The reaction was left stirring under N_2 atmosphere for 24 hours, concentrated and precipitated in cold ether. The yellow precipitate was dissolved and purified with dialysis for 2 days (1 kDa cut off) yielding a yellow powder (5.4 mg, 0.002 mmol, 10 %).

^{1}H NMR (400 MHz, Chloroform-*d*) δ 8.20 (s, 1H), 8.00-7.97 (m, 1H), 7.06 (d, *J* = 8.3 Hz, 1H), 6.77 (s, 2H), 6.68 – 6.51 (m, 4H), 5.22 (s, 1H), 4.37 (dd, *J* = 12.0, 3.3 Hz, 1H), 4.15 (dd, *J* = 12.0, 6.7 Hz, 1H), 4.06 – 3.92 (m, 6H), 3.86 (s, 2H), 3.80 (s, 1H), 3.76 – 3.49 (m, 209H), 3.46 (s, 2H), 3.07 – 2.96 (m, 3H), 2.27 (q, *J* = 7.0 Hz, 4H), 1.33 – 1.15 (m, 68H), 0.90 – 0.83 (m, 7H).

MALDI-TOF MS (m/z) calculated for $C_{158}H_{278}N_3O_{58}PS$: 3162.91; found: 3162.42

Synthesis of vc-MMAE linker

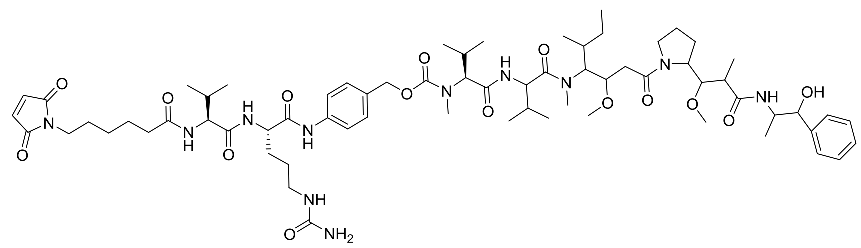

$C_{68}H_{105}N_{11}O_{15}$
L1

MC-val-Cit-PABC-PNP (12.3 mg, 0.0167 mmol, 1 equiv.) was dissolved in dry DMF (0.25 mL) and a solution of MMAE (14.7 mg, 0.0205 mmol, 1.2 equiv.), TEA (3.4 uL, 0.0205 mmol, 1.5 equiv.) and HOBt (3.9 mg, 0.0289 mmol, 1.7 equiv.) in dry DMF (0.25 mL) was added. The reaction mixture was left stirring under N_2 atmosphere for 6 days followed by purification on preperative HPLC (30 % MeCN and 70 % MQ water to 100 % MeCN over 20 min followed by isocratic at 100 % MeCN for additional 2min) with a retention time at 8.7 min, yielding the product as a white powder (10.3 mg, 0.008 mmol, 47 %).

Purity of the isolated product was determined with analytical HPLC (Method: 5 % MeCN to 100 MeCN over 15 min, isocratic for to 20 min.)

HRMS (ESI+) *m/z* calculated (calcd.) for $C_{68}H_{105}N_{11}O_{15}$ + H^+: 1316.7873; found: 1316.7865, calcd. for $C_{68}H_{105}N_{11}O_{15}$ + $2H^+$: 658.8973; found: 658.8971; calcd. for $C_{68}H_{105}N_{11}O_{15}$ + Na^+: 1338.7692; found: 1338.7688; calcd. for $C_{68}H_{105}N_{11}O_{15}$ + K^+: 1354.7432; found: 1354.7427;

HPLC trace of isolated compound:

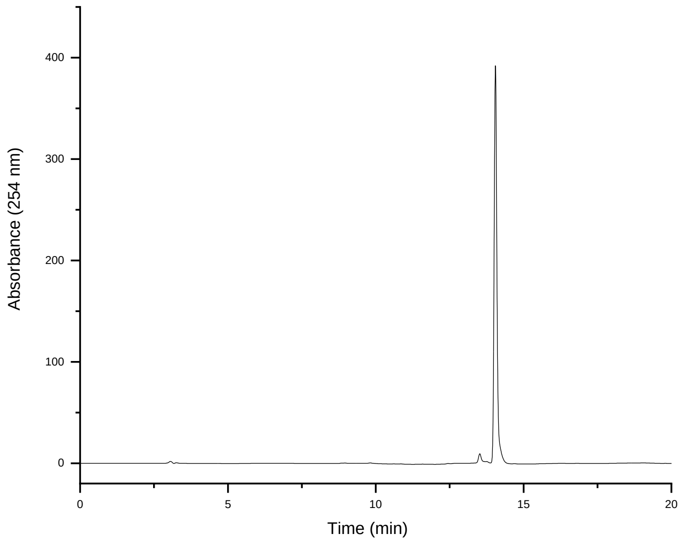

Figure S1. HPLC trace of the isolated product; **L1.**

II. Biological methods

Preparation of antibody-drug conjugates. Monoclonal anti-Fluorescein antibody (> 3 mg/ml) in 500 mM sodium borate/50 mM NaCl PBS buffer, pH 8.0, was treated with TCEP (25 μM final, 3 equiv.) at 37 ℃ for 2 h. The reduced mAb was cooled to 4 ℃ followed by addition of mal-VC-PAB-MMAE linker (50 equiv.) and left overnight for conjugation. The conjugates were spin filtered through Amicon filters (30kDa cut off, regenerated cellulose) followed by gel filtration through a NAP-5 column (Shephadex G-25 DNA grade). Protein concentration where determined with UV-vis λ_{max} 280 nm and drug load was determined with MALDI-TOF-MS.

MALDI-TOF MS (m/z) of unmodified mAb: 149.627 kDa; MALDI MS of modified mAb: 151.037 kDa. The average DAR was calculated to 1.

Binding of receptors with anti-fluorescein antibodies. Solutions of fluorescein and receptors **R3** and **R4** at 100 nM where combined in a microplate with a serial dilution of anti-fluorescein antibody to give final concentrations from 2 nM to 300 nM. For each sample, fluorescence was measured in a plate reader (PerkinElmer EnSpire 2300 Multilabel Reader) (λ_{ex}: 488 nm, λ_{em}: 520 nm) and compared to controls where antibody was not added. The ratio of fluorescence between the sample and the control was plotted. The experiment was reproduced 3 times.

Cell culture. In general, cells were cultivated at 37° C in humidified air with 5% CO_2. After resuscitation, cells were cultured for at least 3 passages before inclusion in experiments unless otherwise specified.

MOLT-4 cell line. Cells were cultured in RPMI 1640 (Sigma R0883) supplemented with 10% FBS (Sigma F7524) , 1% penicillin/streptomycin (Sigma P0781) and 2 mM L-glutamine

(Sigma G2150). Media was changed every 2-3 days and cell concentration was kept between $3x10^5$-$1.5x10^6$ cells/mL. To monitor cultures, cells were stained with Trypan blue (T8154) and counted using a cell counter.

HAP-1 cell line. Cells were cultured in Iscove Modified Dulbecco Media supplemented (Sigma I3390) with 10% FBS, 1% penicillin/streptomycin and 2 mM L-glutamine. Cultures were split upon reaching 70% confluence and media was exchanged every 2-3 days as needed.

PBMC isolation. Human blood from anonymous donors was obtained at the blood bank at Aarhus University Hospital. The blood was diluted 1:1 in PBS (D8537) and 30 mL were overlayed on top of 20 mL Ficoll® Paque Plus (GE Healthcare GE17-1440-03). After centrifugation for 35 min at 400 g, the PBMC layer was separated and washed with 2x20 mL of PBS (10 min, 200 g). If they were not used right away, PBMCs were cryopreserved in FBS supplemented with 10% DMSO.

Negative $CD4^+$ T cell isolation. PBMCs were suspended in 500 μL of isolation buffer (PBS, 0.1% HSA, 2 mM EDTA) at a concentration of 10^8 cells/mL. Afterwards, 100 μL of FBS and then 100 μL of antibody isolation mixture were added. The mixture was incubated at 4° C for 20 min, upon which it was added 500 μL of Dynabeads (ThermoFisher 11352D) (previously washed with 500 μL of isolation buffer) and incubated at room temperature for 15 min with gentle stirring. Finally, 4 mL of isolation buffer were added and the tube was placed on the DynaMag-5 magnet (ThermoFisher 12303D) for 2 minutes before collecting the supernatant. This was repeated two times, the collected fractions were put on the magnet for 2 minutes to remove extra particles. Isolated cells were cryopreserved in FBS supplemented with 10% DMSO at $5x10^6$ cells/mL.

The resulting cells were analyzed by flow cytometry (Guava easyCyte™) alongside with non-isolated PBMC. Briefly, 200 μL of samples at 10^6 cells/mL were stained for 10 minutes at room temperature with 1 μg of a FITC-labelled human antiCD4 antibody (ThermoFisher 11-0049-42) in PBS (2% FBS). Results indicated purity of $CD4^+$ cells higher than 90%, which was not increased by performing the isolation procedure again on the same sample.

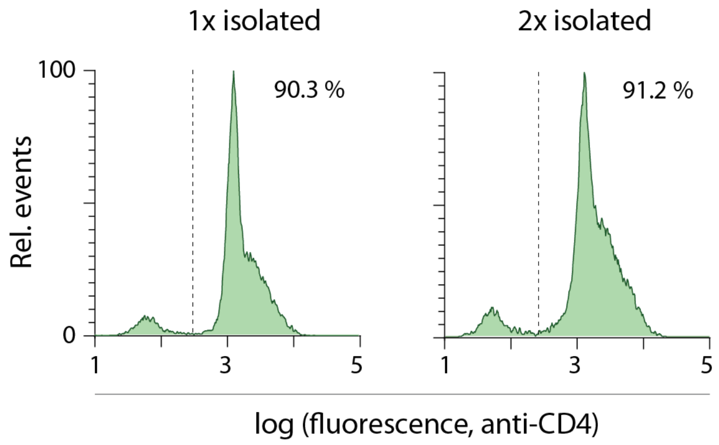

Figure S2. Purity of CD4+ T cells is not increased after two successive isolations from PBMC.

CD4$^+$ T cells culture. After resuscitation, cells where washed once to remove traces of DMSO and suspended at 1.5x10^6 cells/mL in RPMI 1640 media supplemented (10% FBS, 1% penicillin/streptomycin, 2 mM L-Glutamine and 50 U/mL of human interleukin-2 (Roche HIL2-RO). Cells were then combined with Dynabeads T-activator CD3/CD28 (ThermoFisher 11131D) at a 1:1 ratio bead/cell. Cells were kept between 5x10^5 and 2.5x10^6 cells/mL for up to 9 days after activation.

Incorporation of receptors into MOLT4 cells. Cells (4x10^5 cells/mL) were combined with receptors at different concentrations (from DMSO stock solutions) and incubated for 1 h at 37° C in complete media. Control cells were only treated with the equivalent amount of DMSO (0.5%). Afterwards, cells were washed with PBS (300 g, 5 min) and analyzed. For microscopy, cells were stained with Hoescht (ThermoFisher 62249) dye (1:2000 dilution in PBS) for 15 min at room temperature. Then washed with PBS (300 g, 5 min) and imaged at the fluorescence microscope (Zeiss Observer.Z1). For cytometry, cells were washed with cold PBS (2% FBS) twice, fixed with 0.9% formic acid and kept at 4° C until fluorescence was measured at the flow cytometry (at least 10^4 events per sample). The experiments were reproduced 3 times with 3 replicates each time.

Blocking of cell-surface receptor. Cells (4x10^5 cells/mL) were combined with receptor **R2** at 10 μM (from DMSO stock solutions) and incubated for 2 h at 37° C in complete media. Afterwards, cells were washed with PBS (300 g, 5 min) and treated with 0,5 μM of anti-fluorescein antibody in ice cold PBS (2% FBS). Control cells were treated only with PBS (2% FBS). Cells were kept on ice until imaged by confocal laser scanning microscopy (CLSM) (Zeiss LSM 700). The microscope settings were fixed on the sample without the antibody treatment and kept the same for the sample with the antibody treatment.

Percentage on cell surface. MOLT-4 cells (4x105 cells/mL) were combined with receptors at 10 μM (from DMSO stock solutions) at different starting times so that the final incubation times were 4h, 1h and 20 min. Afterwards, all samples were washed with cold PBS (300 g, 5 min, 4o C) and re-suspended in cold PBS (with 2% FBS). Then cells were treated with antiFITC antibody at 0.1 mM. Control samples were not treated with the antibody. Samples were kept on ice until measured at the flow cytometer (Novocyte QuanteonTM). The percentage on the cell surface at each time point was estimated as the percentage of fluorescence quenched between samples treated with antibody and the controls not treated with antibody. The experiment was reproduced 3 times with at least 3 replicates each time.

Internalization of antibodies. MOLT-4 or human $CD4^+$ T cells ($4x10^5$ cells/mL) were combined with receptors at 10 μM (from DMSO stock solutions, final DMSO concentration 0.2%) for 2h at 37° C. Then cells were washed with PBS (300 g, 5 min) suspended in complete media and incubated for 1 h with antibodies at 0.2μM (TRITC- anti-fluorescein or human antiCD4-FITC). Afterwards, cells were washed with PBS, stained for 15 minutes at room temperature with Hoescht (1:2000 dilution in PBS) and washed with PBS before imaging at CLSM.

Blocking of TRITC- anti-fluorescein antibody internalization. MOLT-4 ($4x10^5$ cells/mL) were combined with receptors at 10 μM (from DMSO stock solutions, final DMSO concentration 0.2%) for 2h at 37° C. Then cells were washed with PBS (300 g, 5 min) and suspended in complete media in the presence of the antibody (0.2 μM) either at 37^oC or at 4^oC for 20 min. Afterwards cells were washed with PBS, stained for 15 minutes with Hoescht and washed with PBS before imaging at CLSM. Samples treated at 4° C were kept at that temperature throughout all washing/staining steps.

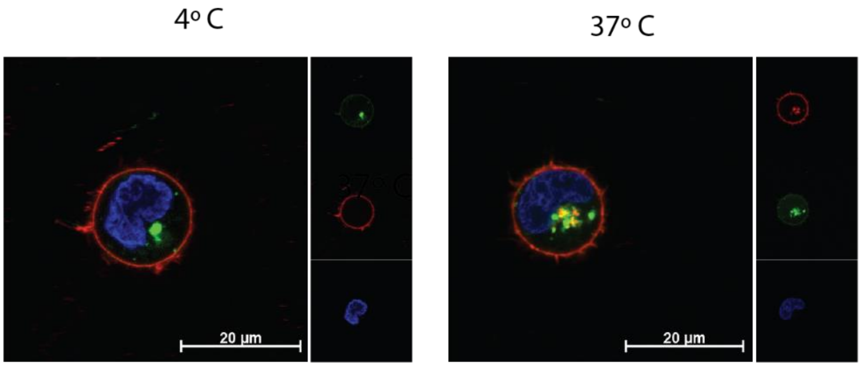

Figure S2. CLSM images of MOLT-4 cells after treatment with receptor 2 and TRITC- anti-fluorescein at 37° C (right) or at 4° C (left).

Uptake of antiFITC-TRITC antibody. Cells ($4x10^5$ cells/mL) were combined with receptors at 10 μM (from DMSO stock solutions) and incubated for 2 h at 37° C in complete media. Control cells were only treated with the equivalent amount of DMSO (0.2 %). Afterwards, cells were washed with PBS (300 g, 5 min) and incubated for 20 min or 4 h with antiFITC-TRITC antibody (0.2 μM). Samples were washed with PBS (2% FBS), fixed with 0.9% formic acid and kept at 4° C until analyzed by flow cytometry (Novocyte Quanteon™) (at least 10^4 events per sample). The experiment was reproduced 3 times with 3 replicates per time.

Incorporation of receptors on PBMC and antibody uptake. PBMC were resuscitated into warm complete RPMI1640 media and washed once with media (300 g, 5 min) and left to incubate at 37° C. After 2h, cells ($5x10^5$ cells/mL) were incubated with receptors **R1**-**4** (from DMSO stock solutions) at 10 μM for 2h at 37° C. Control cells were added no receptor but equivalent amount of DMSO. Then cells were washed with PBS (300 g, 5 min) and changed back to media. A set of samples for each receptor was treated with 0.2 μM of TRITC- anti-fluorescein antibody (from PBS stock solutions) for 1h at 37° C. Other samples were treated just with PBS. Afterwards cells were washed with PBS (2% FBS) and analyzed by flow cytometry (at least $3x10^4$ events PBMC). For each sample, monocyte and lymphocyte populations were gated on the FSC vs SSC plot and the histogram distribution of fluorescence of receptor or antibody was analyzed for each population respectively.

Potency of ADC treatment. Cells at normal seeding densities were combined with receptors at 10 μM (from DMSO stock solutions) and incubated for 2 h at 37° C in complete media. Control cells were only treated with the equivalent amount of DMSO (0.2 %). Afterwards, cells were washed with PBS (300 g, 5 min) and suspended in complete media. Then, each of the receptor-modified cells and control cells were combined with a ADCs (from PBS solutions). A control were no ADC was added was prepared for each set of samples. For

receptor **R1** and MOLT-4 cells, samples in the presence of 25 μM fluorescein were also prepared.

Cells were cultured normally for 72 h upon which viability was measured using a metabolic assay. Briefly, PrestoBlue® (ThermoFisher A13262) reagent was diluted 1:10 in the cell media and incubated for 1h upon which fluorescence of resorufin was measured at λ_{ex}: 536 nm λ_{em}: 619 nm. The experiments were reproduced 3 times with at least 2 replicates each time.

Dose response curves on synthetic receptor feed concentration. MOLT-4 cells were seeded in 96-well plates with a round bottom at normal seeding densities in complete media. Then they were combined with a dilution series of R1, R2 and R4, (from PBS stock solutions, equalized to contain the same DMSO content). Control samples without receptor were also prepared. After 2 h of incubation at 37° C, the cells were washed with PBS (300 g, 5min) and changed back to complete media and treated with 100 nM of ADC. After 72 h, cell viability was assessed with the PrestoBlue® metabolic assay. Viability was assessed against controls without receptor or antibody treatment. The experiments were reproduced 3 times with at least 2 replicates each time.

Spheroid formation and culture. 3D cell cultures were prepared by growth of HAP-1 cells ($2x10^3$-$2x10^4$ cells/well in complete media) in ultra-low attachment spheroid plates (Corning 4515). Cells were left to grow in complete media, which was exchanged every 2-3 days. 3D cultures were monitored by microscopy.

ADC treatment in 3D cell culture. HAP-1 cells ($2x10^4$ cells/well) were seeded in low attachment spheroid plates and grown in normal conditions. After 96 h, cell spheroids were treated with 10 μM of receptor **R2** for 2 h or 24 h at 37° C. After washing with PBS, spheroids were incubated in complete media in the presence of ADC (150 nM). Control spheroids were incubated just in complete media. After 48 h, spheroids were imaged at the

fluorescence microscope after performing a live dead stain. Before, spheroids were washed with PBS and incubated at room temperature with fluorescein diacetate (Sigma F7378) or propidium iodide (Sigma P4170) (4 and 6 μg/mL respectively) for 20 min.

Statistical analysis. Where reported, statistical significance was evaluated with a two-way ANOVA with the Sidak's multiple comparisons test performed in the software Graphpad Prism®.

III. ¹H and ¹³C NMR spectra

^{1}H-NMR and ^{13}C-NMR of structure a

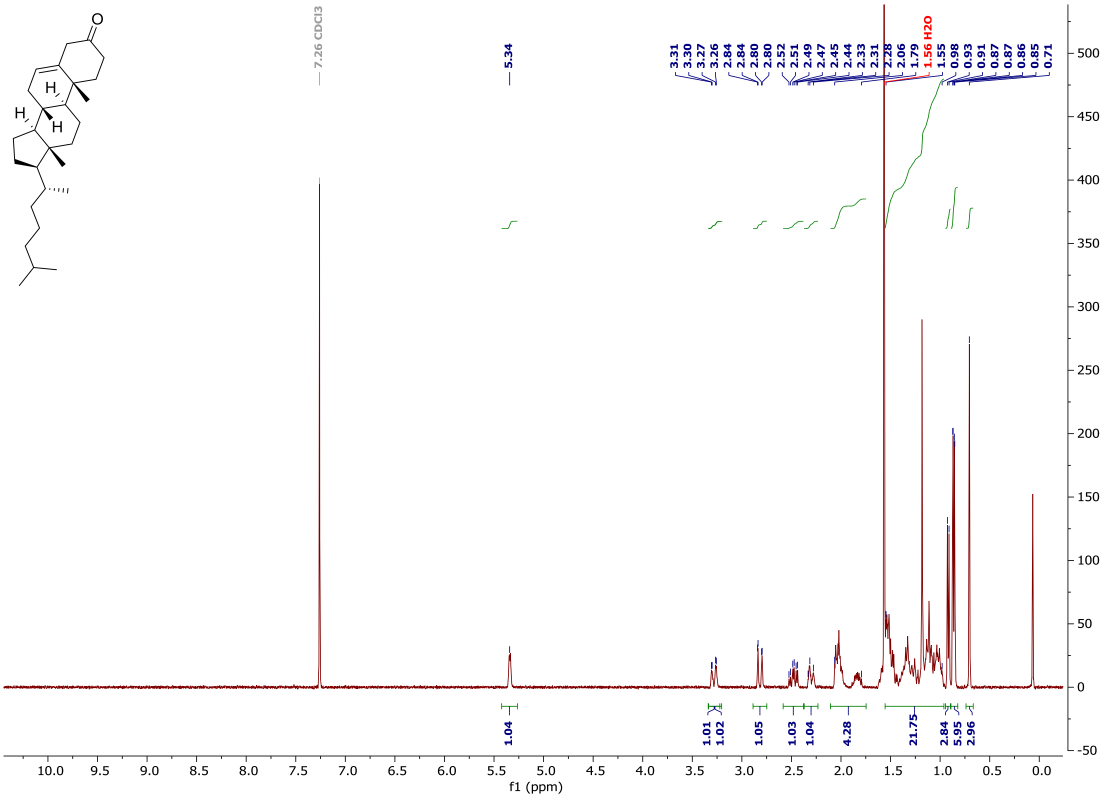

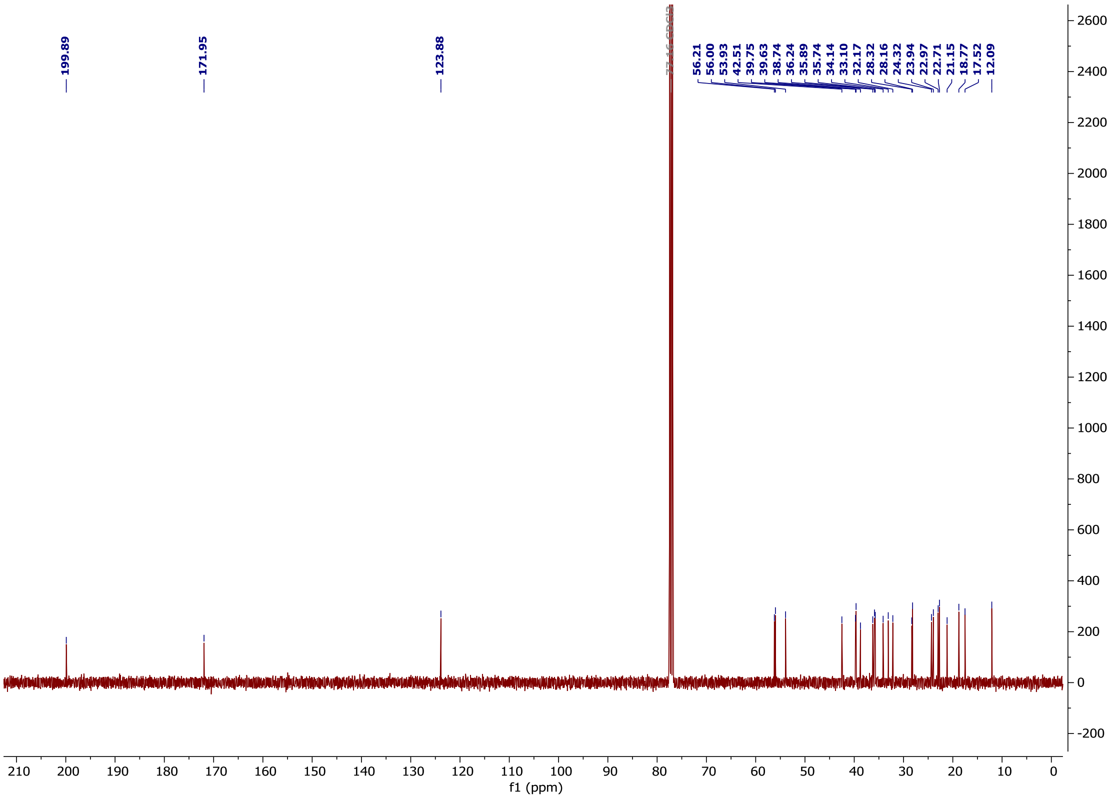

^{1}H-NMR and ^{13}C-NMR of structure b.1

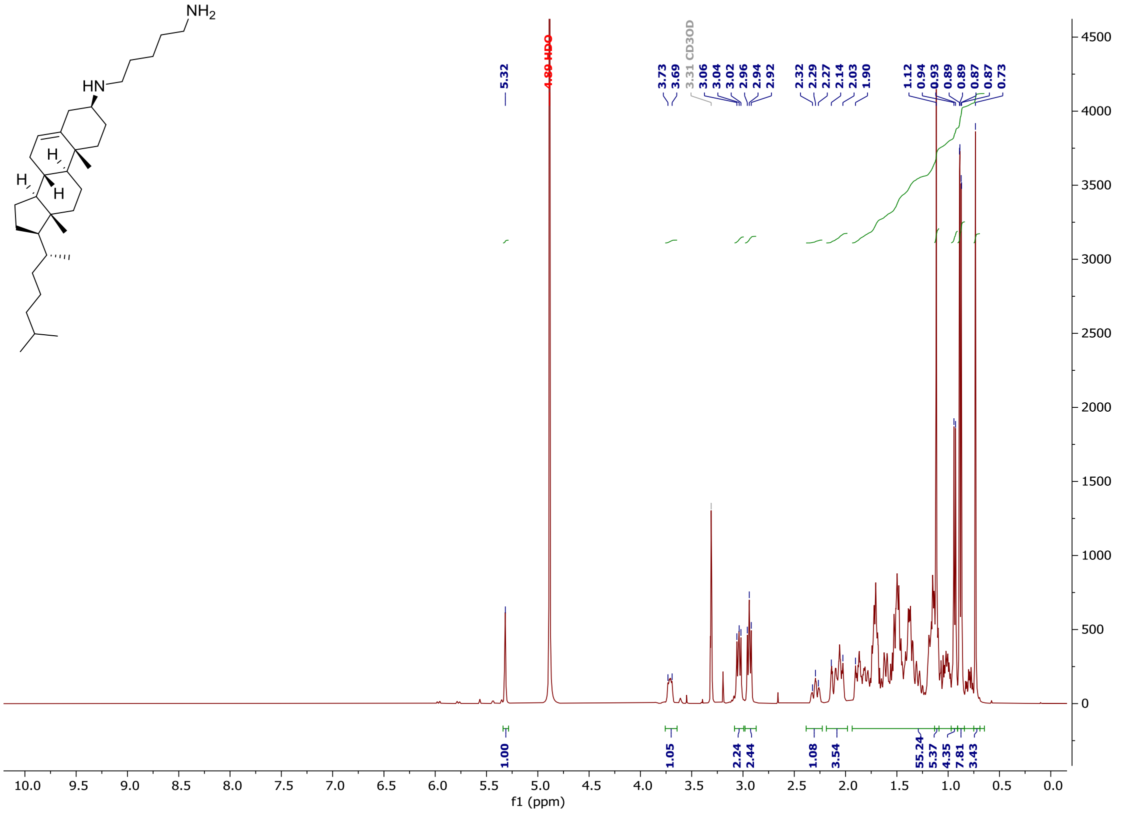

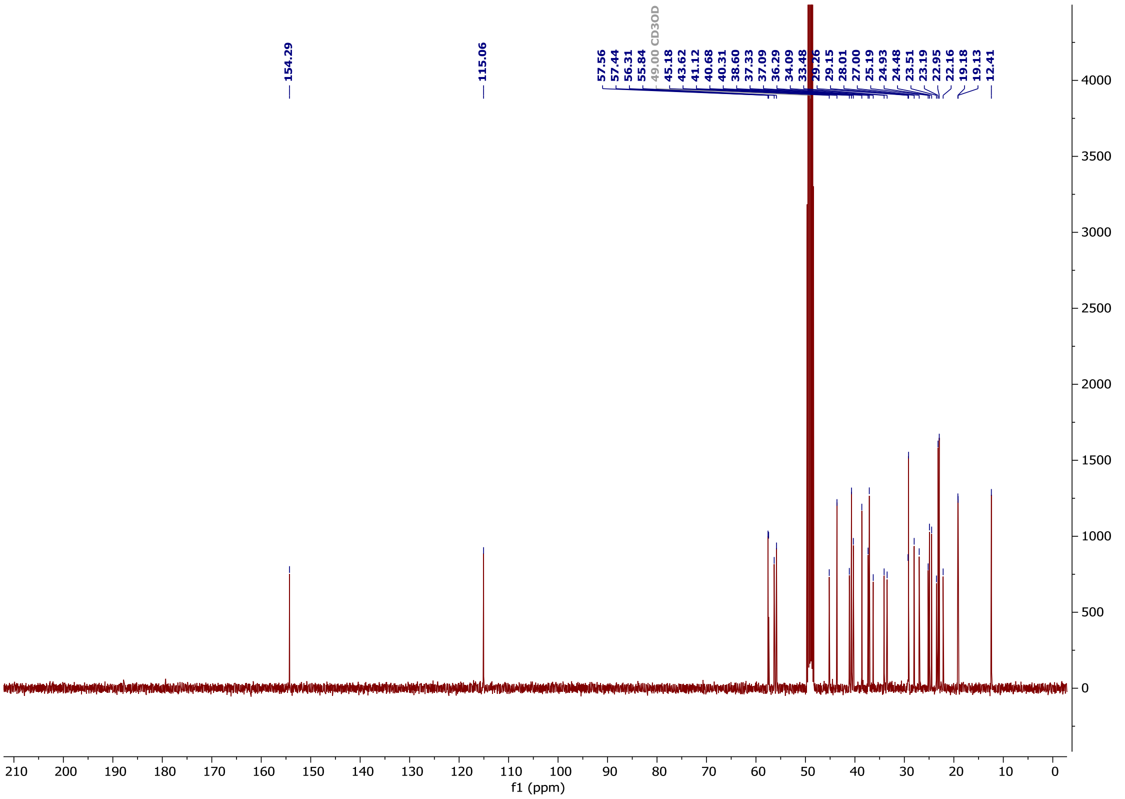

^{1}H-NMR of structure b.2

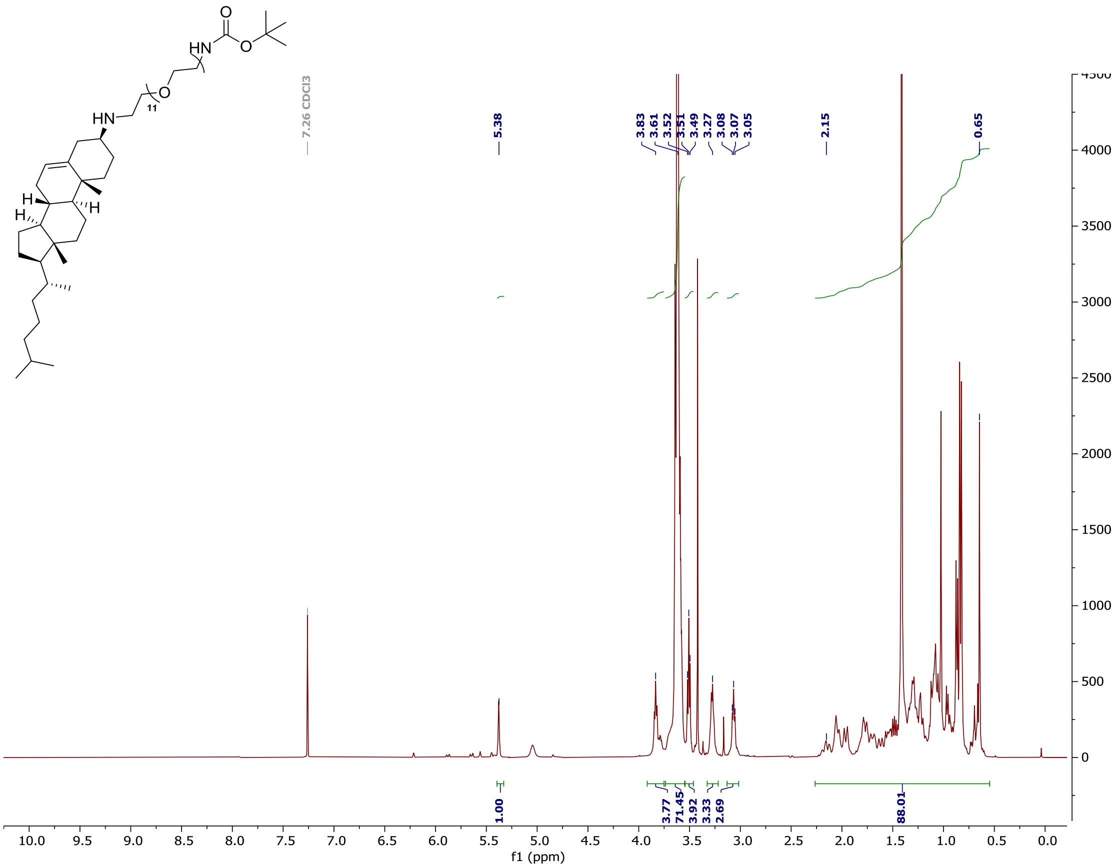

^{1}H-NMR of structure b.3

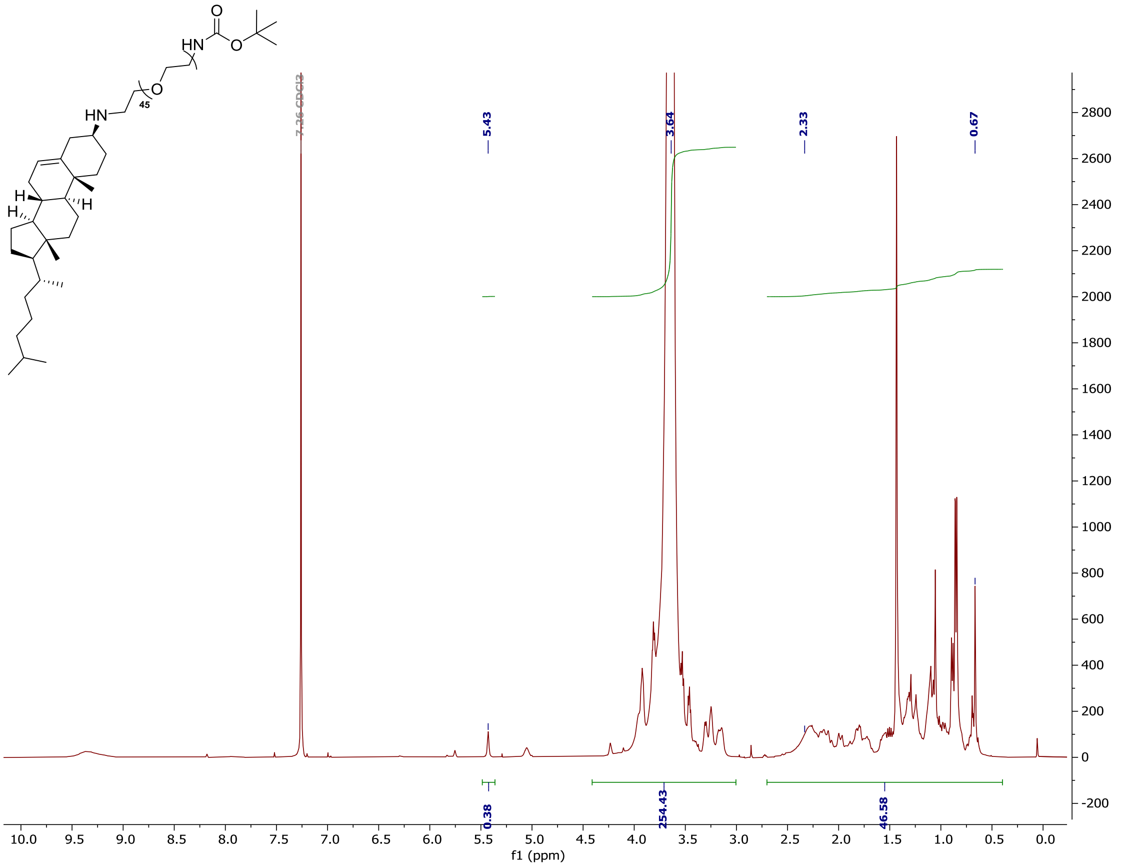

^{1}H-NMR of structure R1

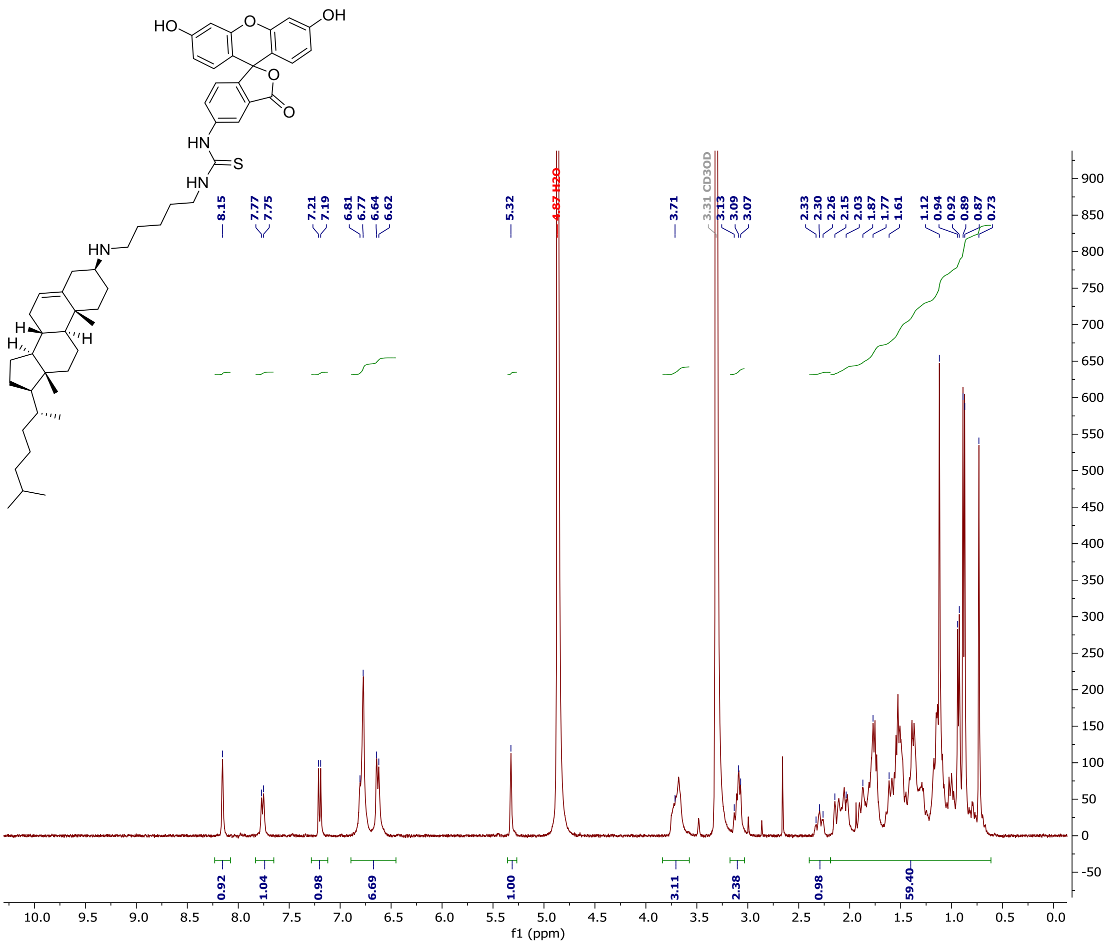

^{1}H-NMR of structure R2

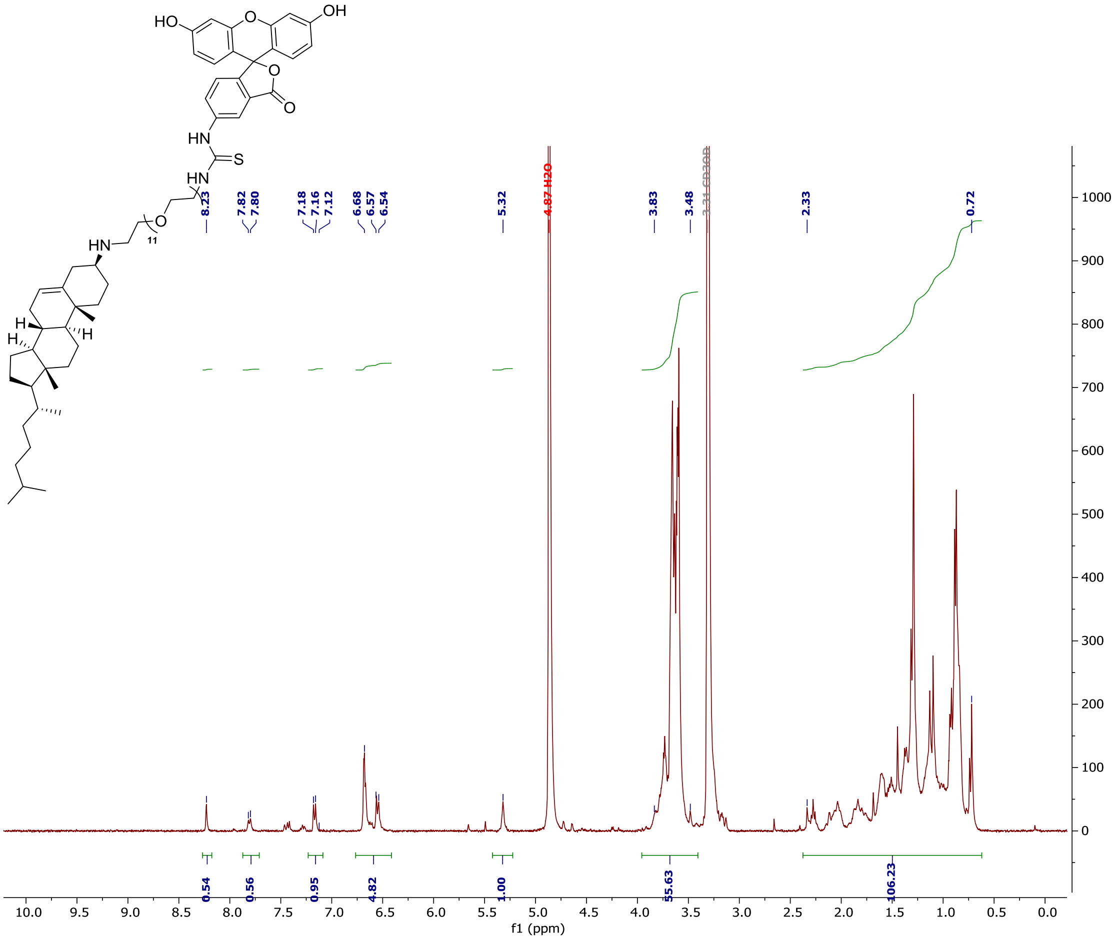

^{1}H-NMR of structure R3

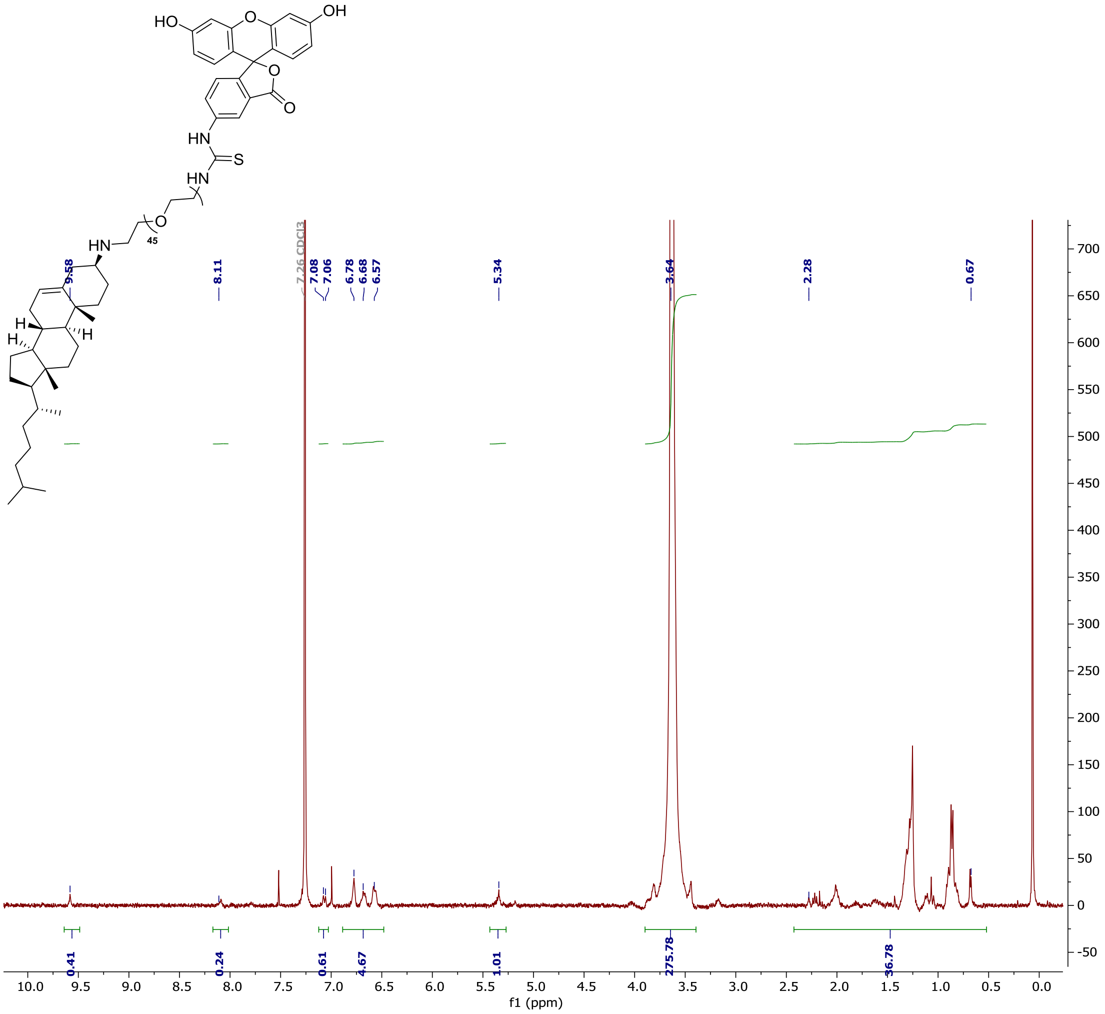

^{1}H-NMR of structure R4

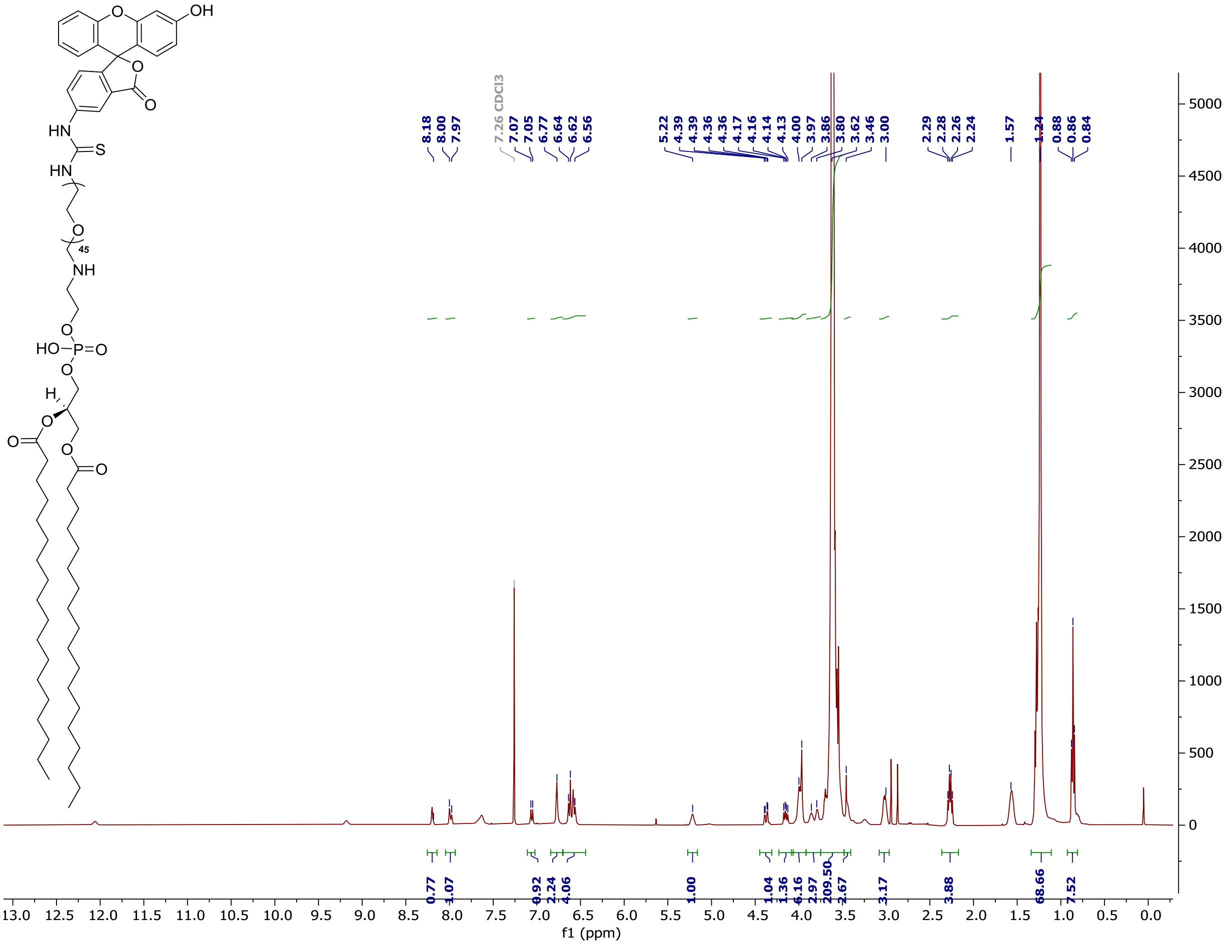

Supplement: Supplementary file 1 — Supporting Information [file ADVS-7-2001395-s001.pdf]
